# Qualification of Dentists in Prussia

**Published:** 1845-03

**Authors:** 


*Qualification of Dentists in Prussia.*—By a government order, dated the 29th April, 1835, directed to the different governments and medical colleges of the kingdom, it is decreed, that no person shall be admitted to examination for a license to practice as a dentist, who, in addition to his testimonials of having acquired practical dexterity in his art with a licensed and practising dentist, shall not adduce proofs of being in one or other of the following conditions:

1. Of being a licensed physician or surgeon.
2. Of having served three years as surgeon in the army.
3. Of having obtained such knowledge and skill as are necessary for a surgeon in a regular attendance at places of public education.

In reference to this last condition, he must bring testimonials of having attended, during a curriculum of two years, lectures on anatomy; the theory of medicine; general, special, and operative surgery; a chirurgical clinic; and, when practicable, particularly on dental surgery.—*Rust's Mag.* B. 45, *Heft* 1, 1835.

